# Exploring photosensitization as an efficient antifungal method

**DOI:** 10.1038/s41598-018-32823-2

**Published:** 2018-09-27

**Authors:** César Espinoza, Miriam C. Rodríguez González, Guillermo Mendoza, Alberto Hernández Creus, Ángel Trigos, José J. Fernández

**Affiliations:** 1 Instituto Universitario de Bio-Orgánica Antonio González (IUBO AG), Centro de Investigaciones Biomédicas de Canarias (CIBICAN), Universidad de La Laguna, Universidad de La Laguna (ULL), Avenida Astrofísico Francisco Sánchez 2, 38206 Tenerife, Spain; 2 Laboratorio de Alta Tecnología de Xalapa, Universidad Veracruzana, Calle Médicos 5, Col. Unidad del Bosque, 91010 Xalapa Enríquez, Veracruz Mexico; 3 Área de Química Física, Departamento de Química, Instituto de Materiales y Nanotecnología (IMN), Universidad de La Laguna (ULL), Avenida Astrofísico Francisco Sánchez s.n., 38200 Tenerife, Spain; 4 Facultad de Ciencias Agrícolas, Universidad Veracruzana, Circuito Gonzalo Aguirre Beltrán s.n., Zona Universitaria, 91090 Xalapa, Veracruz Mexico; 5 Departamento de Química Orgánica, Universidad de La Laguna (ULL), Avenida Astrofísico Francisco Sánchez s.n., 38206 Tenerife, Spain

## Abstract

Lipid bilayers containing ergosterol show signs of destruction when they are treated with singlet oxygen, due to the conversion of ergosterol into its peroxy derivative. Applying this previous knowledge, an antifungal method was explored using *Candida tropicalis* as model, and membrane permeation under photosensitization conditions became evident. These data were complemented through AFM images of artificial lipid bilayers, using cholesterol or ergosterol as structural sterols, showing their corresponding morphologies at the nanoscale. Based on these results, an antifungal method was developed, which shows evidence of the extent of membrane permeation during photosensitization. Such photosensitization offers an effective alternative treatment, especially in membranes with a high ergosterol content, suggesting that this procedure constitutes an easy and efficient antifungal method.

## Introduction

The cell membrane is undoubtedly one of the most complex and important structures in unicellular organisms. Membranes are made up of a lipid bilayer, in which a large number of proteins with diverse important functions are immersed, embedded, or attached, or otherwise associated with it. The lipid bilayer is mainly formed from three classes of amphipathic lipids: phospholipids, glycolipids, and squalene-derived metabolites (hopanoids in prokaryotes or sterols in eukaryotes). These metabolites act as integrating elements that improve packaging and render an appropriate consistency^1–5^. They are also a differentiating factor when bacterial, plant and animal cells are compared with those of fungal origin. In the previous cases, hopanoids or cholesterol are the predominant sterols, while ergosterol is present in fungal cells. Sterols are biological molecules representing from 20 to 50% of a cell membrane, and they are essential for maintaining the proper structure and function of eukaryotic cell membranes^3^. The design of some selective antifungal treatments is based on this different composition and reactivity of ergosterol and cholesterol^6^. In this way, the well-known photo-oxidation reaction of ergosterol by singlet oxygen can be used for selective elimination of unicellular fungi^7–10^. Thus, ergosterol has been easily oxidized to its peroxide form in the presence of molecular oxygen and photosensitizers such as Eosin Y. This is an excellent starting point for the development of possible antifungal strategies^10–13^.

Due to the chemical and structural diversity of cell membranes, a model membrane system is necessary to analyse biological processes at cellular level. Model membrane systems have been extensively studied in order to improve the understanding of structural properties and organization of biomembranes, and even the activity of surfactants^14^ or proteins^15^ on them. Different alternatives such as liposomes, Langmuir-Blodgett films^16^ or supported lipid bilayers (SLBs) have been proposed as model membranes. Among them, SLBs are easy to prepare in a wide range of substrates, leading to the formation of a planar, stable and well-organized nanostructure^17^. Although the solid substrate partially influences the properties of SLBs, both thermodynamic and structural properties are retained, facilitating study with localized techniques.

Nanometre-level techniques such as atomic force microscopy (AFM) are suitable to characterize SLBs and their properties with nanometric resolution^18^. The advantage of AFM is the possibility to control environmental conditions. SLBs can be characterized in liquid media, for which, extremely flat surfaces are required. Substrates like ITO^19^, highly-oriented pyrolytic graphite (HOPG)^20^ or mica^21^ have been explored as substrates to support SLBs.

**Figure 1:**
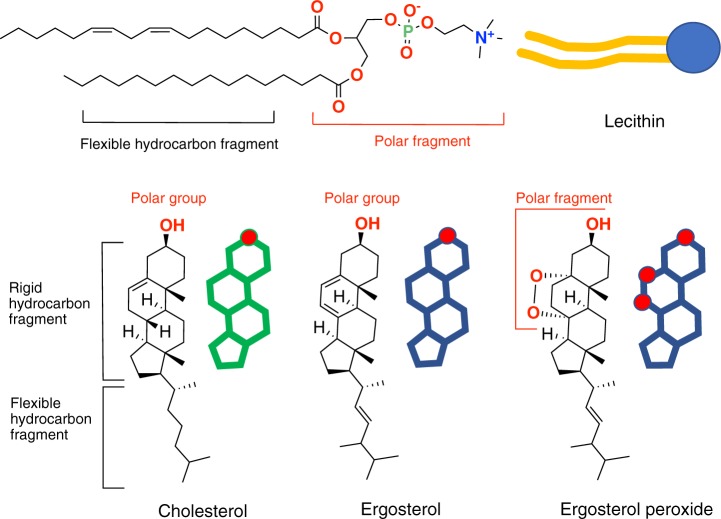
Chemical structures and schematic representation for cholesterol, ergosterol, ergosterol peroxide and lecithin.

In the present study, we have studied the preparation of lecithin-SLBs with cholesterol or ergosterol incorporated into their structures (Fig. 1). The conservation or destabilization of these structures in the presence of photosensitizers and oxygen was explored. Finally, taking advantage of the different sterols in their membranes, the response of two pathogenic microorganisms, *Candida tropicalis* and *Escherichia coli* (as control), was tested.

## Experimental Section

### Materials

L-α-phosphatidylcholine (L-α-lecithin) from soy type II-S 14–23%, cholesterol (>95% HPLC), ergosterol (>95% HPLC), chloroform (≥99.8% ACS spectrophotometric grade), Chromasolv methanol (HPLC grade ≥99.9%) and Eosin Y(≥75%) were all purchased from Sigma-Aldrich (Germany). All experiments were performed in 20 mM Hepes buffer (Sigma-Aldrich ≥99.5%), 20 mM MgCl_2_ (Janssen Chimica >98%) and 150 mM NaCl (Merck 99.5%) at a pH of 7.4, dissolved in water (HPLC grade, Scharlau) and sterilized in a vacuum filtration system, using a PES membrane filter (0.2 μm pore size, Sartorius, Göttingen, Germany).

### AFM study of membrane prototypes

The bilayer morphology was characterized by AFM images in liquid. Bilayers were deposited on freshly-cleaved mica substrates. HEPES (50 μL) was added onto the disc prior to being placed into the AFM liquid cell. The surface was mapped in HEPES at room temperature using a Nanoscope V Controller (Bruker). Images were acquired in peak force tapping mode. The AFM probes used in these studies were silicon nitride (NP-C, Bruker) with a nominal tip radius of 20–60 nm and a cantilever spring constant that ranged between 0.14–0.26 N/m.

Lecithin and cholesterol or ergosterol were dissolved in a chloroform:methanol (3:1) mixture to a 3 mM concentration. The solvent was then evaporated, obtaining a thin film on the walls of the tube. Next, the dried phospholipid films were hydrated with buffer solution at a temperature above the transition temperature of the lipid. A concentration of around 500 μM was reached. The tubes were subjected to cycles of vortex mixing (1 min) and heating (20 s) at around 60 °C. The vesicle suspensions were placed in an ultrasound bath for 30 min to finally obtain mainly unilamellar vesicles.

**Figure 2:**
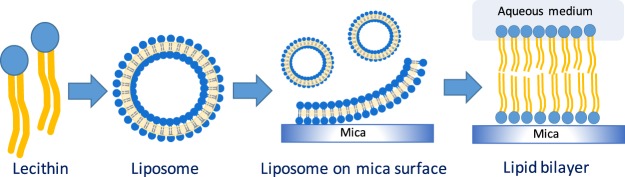
Process for obtaining the lipid bilayer on mica.

To obtain supported membrane systems, 100 μL of this liposome suspension was deposited on a freshly-cleaved mica surface and the temperature was set for 10–20 min above the transition temperature of the lipid. After that, the samples were thoroughly rinsed with buffer solution and always kept hydrated on the mica substrate (Fig. 2).

**Figure 3:**
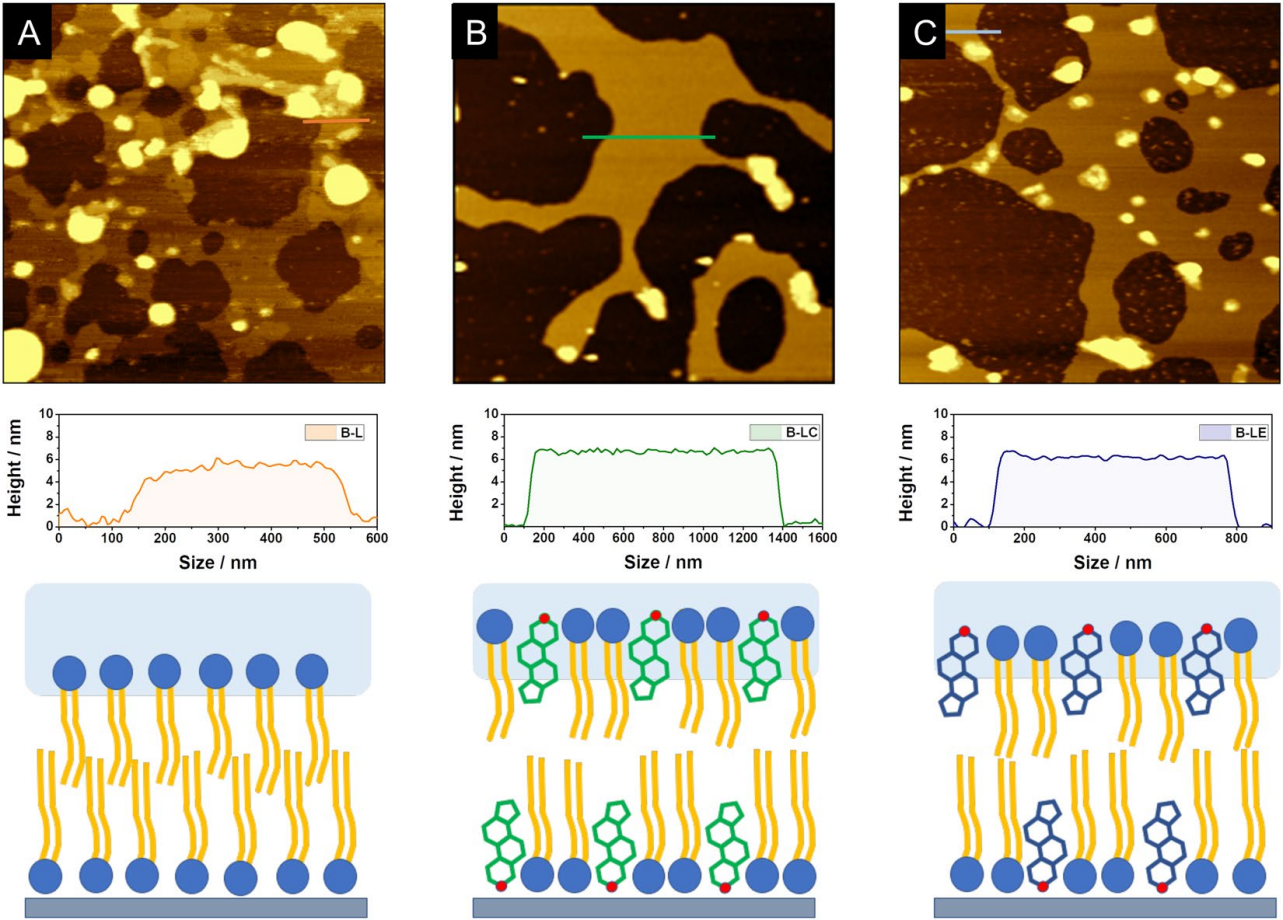
Topographic AFM images (3 × 3 μm) for (**A**) bilayers with only lecithin; (B-L; 5.1 nm); (**B**) bilayers structured with lecithin and cholesterol 40% (B-LC; 6.3 nm), and (**C**) bilayers structured with lecithin and ergosterol 40% (B-LE; 6.1 nm) on the mica surface. Representative cross-sections and diagrams are included.

Artificial bilayer samples were prepared as indicated in the experimental section, to study the surface properties induced by changes in membrane composition. In our approach, three different types of bilayers were studied, composed of (i) lecithin (B-L), (ii) cholesterol-containing lecithin (B-LC) and (iii) ergosterol-containing lecithin (B-LE). For bilayers with both cholesterol or ergosterol, a percentage of 40% was chosen (near the proportion observed in animal cell membranes), as a reference for comparison. Figure 3 shows the AFM images for (a) B-L, (b) B-LC and (c) B-LE and representative cross-sections.

### Photosensitization of microorganisms

According to the methodologies proposed by Trigos & Ortega-Regules^10^ and Lagunes & Trigos^11^, solutions of Eosin Y (144 μM) and ergosterol (1 mM) were prepared. Additionally, 20 mM Hepes buffer, 20 mM MgCl_2_ and 150 mM NaCl solutions at pH 7.4, saturated with oxygen (medicinal grade) were inoculated with a suspension of 10^5^ CFU (colony forming units) of the pathogenic yeast *C*. *tropicalis* (CECT 1440) or of the bacterium *E*. *coli* (CECT 943), obtained from the Colección Española de Cultivos Tipo (CECT). These solutions were mixed as indicated below and irradiated for 2 h in a photooxidation chamber (30 × 30 cm), using four fluorescent lamps (CFLT4- GE 20 W) as light source, and an oscillator agitator for culture tubes (Rocking Mixer, Scientific). This system has a light emission spectrum between 380–780 nm. All the photosensitization reactions were carried out three times. In order to evaluate the photo-oxidation of *C*. *tropicalis*, four experiments were designed under both light and dark conditions, as detailed below.

#### Procedures with photo-radiation

Two trials were carried out under photosensitization conditions.

- Reaction 1 L (*Growth control*): Using 5.0 mL of Hepes buffer pH 7.4 and 10^5^ CFU of microorganism.
- Reaction 2 L (*Photosensitization treatment*): 4.8 mL of Hepes buffer pH 7.4 and 10^5^ CFU of microorganism, 200 μL of Eosin Y at 144 μM.

#### Procedures without photo-radiation

Identical trials were carried out in dark conditions.

- Reaction 1D (*Growth control*): Using 5.0 mL of Hepes buffer pH 7.4 and 10^5^ CFU of microorganism.
- Reaction 2D (*Negative photosensitization treatment*): 4.8 mL of Hepes buffer pH 7.4, 10^5^ CFU of microorganism, 200 μL of Eosin Y at 144 μM.

All the processes were monitored for photo-oxidation of ergosterol to its peroxide. Once the irradiation time or dark period was finalized (2 h), the reagents and the microorganism biomass were separated by centrifugation for 5 minutes at 5000 rpm, and subsequently extracted with CHCl_3_. The solvent was evaporated *in vacuo* and ergosterol peroxide was determined by ^1^H NMR (600 MHz using CDCl_3_), based on the comparison of the integrals of the vinyl signals (H-6 and H-7) in the B-ring of ergosterol *vs* ergosterol peroxide^11^.

### Photodynamic inactivation and determination of cell viability

To determine the effect on cell viability, after the photosensitization and dark periods, samples of each microorganism (100 μL) from the procedures 1 L, 2 L and 1D, and 2D were inoculated on the media. *C. tropicalis* was incubated on potato dextrose agar (PDA) at 27 ± 2 °C for 72 h (Fig. S1), and *E*. *coli* on Müeller Hinton agar at 35 ± 2 °C for 24 h. Once the incubation period was over, the CFU were quantified using a MiniLight Box Mod. Petite colony counter (Table 1).

**Table 1:** Determination of cell viability for *C*. *tropicalis* and *E*. *coli* after photosensitization treatments.

Treatments	*Candida tropicalis* CFU/mL	% *survival*	*Escherichia coli* CFU/mL	% *survival*
***Photosensitizisation***, ***light conditions***				
1 L	1.5 × 10^5^	98.6 ± 2.5	2 × 10^5^	98.9 ± 1.9
2 L	Not detected	0.0	2 × 10^5^	100.2 ± 6.6
***Photosensitization***, ***dark conditions***				
1D	1.5 × 10^5^	100 ± 0.0	2 × 10^5^	100 ± 0.0
2D	1.2 × 10^5^	80 ± 6.6	2 × 10^5^	96.8 ± 3.2

### Morphological and confocal scanning laser microscopy analysis

After the light and dark photosensitization periods (1–2 L and 1–2 D protocols), samples of *C*. *tropicalis* from under each of the conditions were analysed in optical microscope 100X (Axiostar, Carl Zeiss) and atomic force microscopy (AFM), to check if the morphology of the microorganisms was normal (Fig. S2).

**Figure 4:**
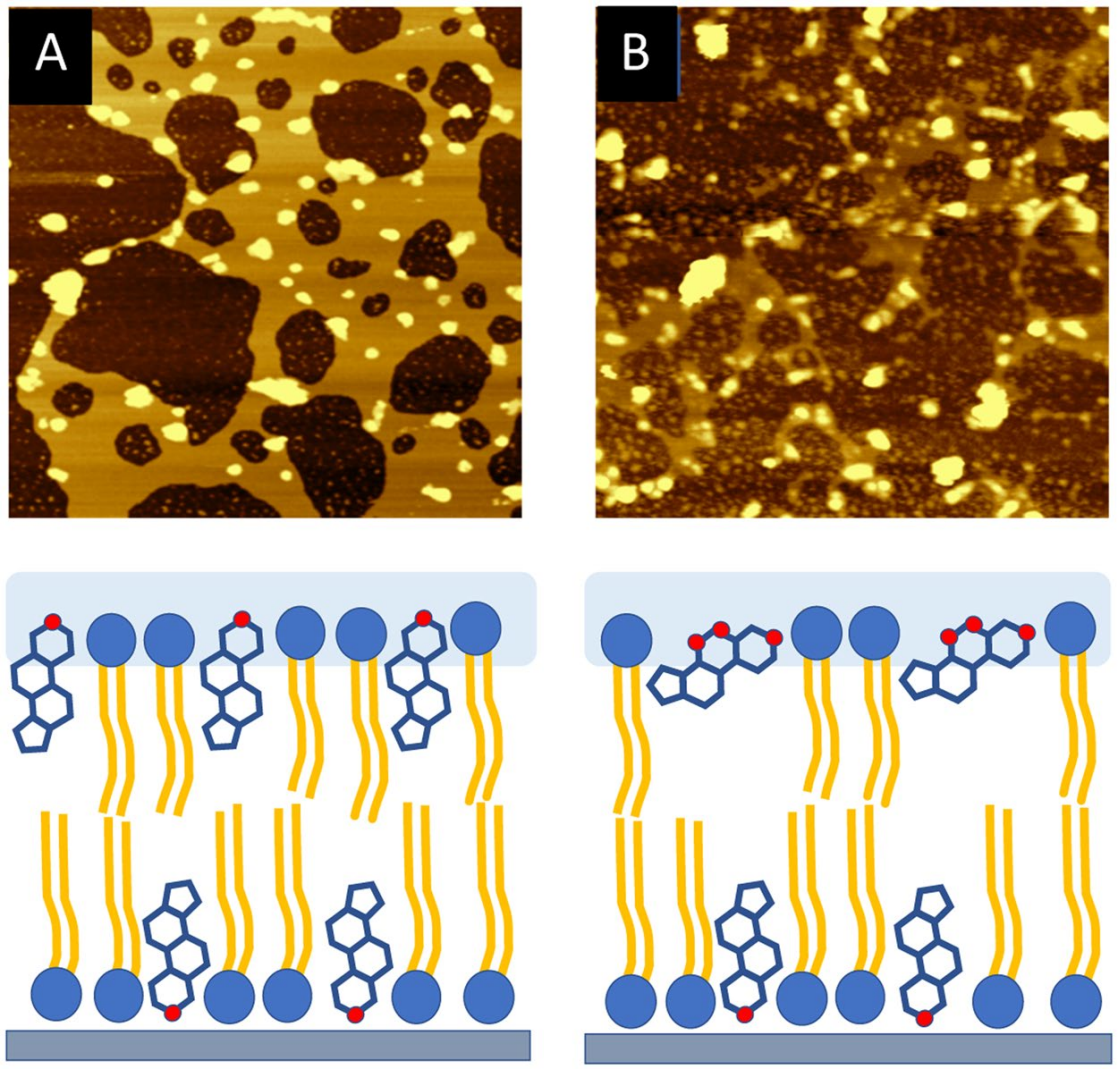
AFM images of bilayer structured with lecithin and 40% ergosterol on the mica surface, (**A**) before and (**B**) after the exposure to photosensitization treatment for 2 h.

To characterize cell morphology and membrane damage under both 2 L and 2D reaction conditions for *C*. *tropicalis* and *E*. *coli*, 1 mg/mL of propidium iodide (PI) was added to the cell suspensions and incubated for 30 min at room temperature in dark conditions. The cells were examined with a confocal scanning microscope (Leica DMI 6000) with lasers for fluorescence excitation (Leica TCS-SP8), using helium green neon laser at 543 nm for PI. The images are represented in Figs 4 and S3.

## Results and Discussion

### Atomic force microscopy study

In the case of B-L, the mica surface became covered with non-uniform aggregates. This is due to the absence of a sterol-type compound in the structure of the bilayer. It is well known that sterols provide the membrane with the necessary consistence and mechanical stability. Thus, as B-L layers are composed of only lecithin, the bilayer structure is neither well-ordered nor uniform. Figure 3A clearly shows the accumulation of material into a second or third layer on top of the bilayer. The height of this bilayer in contact with the surface was 5.1 nm. In contrast, when 40% cholesterol was added during liposome formation, a very well-defined uniform layer was obtained (Fig. 3B), around 6.3 nm high. Finally, Fig. 3C shows the results obtained for the 40% ergosterol-containing bilayer. Interestingly, in this case, a less uniform structure than that of the cholesterol samples was observed in AFM images. It is clear that ergosterol stabilizes the bilayer structure when compared to the B-L samples, although to a lesser extent than in B-LC. As a consequence, a slightly lower height of around 6.1 nm was found for B-LE. This difference between cholesterol and ergosterol has been reported previously^6^. In this type of structure, an increment in the height is observed when cholesterol or ergosterol is mixed with a phospholipid. The increment is greater for bilayers that include cholesterol in their structure, compared with ergosterol, which contains an additional methyl group in the side chain. This result is a direct consequence of the stronger condensing properties of the cholesterol in phospholipid bilayers, compared to ergosterol. Figure 4 shows AFM images after 2 h of 380–780 nm light radiation, in contact with Hepes buffer solution pH 7.4 and Eosin Y at 144 μM, saturated with oxygen.

The experiment was carefully controlled in order to obtain the post-treatment image approximately in the same area where the pre-treatment image was taken. In these conditions, ergosterol is transformed into its peroxide derivative^10,11^. As a result, there is a considerable increase in the number of polar groups in the sterol that unites the lipid bilayer, so it becomes more permeable. The oxidized sterols must accommodate their spatial arrangement in the environment of the membrane, and this in turn also becomes more permeable (Figs 1 and 4).

Shortly before this study, to explore the mechanisms underlying azole resistance in *C*. *tropicalis*, fifty-two clinical isolates were collected and antifungal susceptibility tests performed^22^. The results did not reveal a significant change in their overall sterol levels, but the mean ergosterol content of the resistant group was higher than that of the susceptible group. Additionally, the fluconazole-resistant isolate showed a 1.2-fold higher ergosterol content than its matched fluconazole-susceptible isolate. Furthermore, some isolates were multiresistant to two or three types of azole antifungals. Their ergosterol contents were slightly higher than the isolates that were only resistant to fluconazole or itraconazole. Thus, as ergosterol levels increase in the membrane, these microorganism strains show a greater resistance to this type of antifungal agents^23^. These high ergosterol levels can be harnessed to overcome this resistance through a photosensitization process.

Exploring this alternative for antifungal treatment by photosensitization, we chose *C*. *tropicalis* as a fungus model organism with ergosterol in its membrane, to submit it to our method with singlet oxygen and analyse its viability, using *E*. *coli* cultures as control.

### Photosensitization of microorganisms and cell viability experiments. Proof of concept

On the basis of the AFM results regarding the photo-oxidation of ergosterol and its consequences on the cell membrane, *C*. *tropicalis* and *E*. *coli* were cultured under all the photosensitization conditions, and the samples then carefully analysed. From these experiments, it was possible to demonstrate the fungicidal effect of the 2 L procedure, where there was no growth of *C*. *tropicalis* after 2 h of irradiation and subculture on PDA to 25 ± 2 °C, for 72 h (Fig. S1). The amount of viable *C*. *tropicalis* at the beginning of the photosensitization procedures was 10^5^ CFU/mL, and its viability was not affected after photo-treatment (Reaction 1 L), obtaining 1.5 × 10^5^ CFU/mL. Similarly, the presence of the photosensitizer (Reaction 2D) in dark conditions did not affect its development on PDA plates (see Table 1).

After the photosensitization period, samples of *C*. *tropicalis* from reactions with and without photosensitizer (1 L and 2 L) were observed under 100 X microscope and AFM (Fig. S2). Neither morphological changes in the yeasts, nor any alteration in their structure were detected, in spite of the non-viability of *C*. *tropicalis* cultures in samples after light-treatment in the presence of oxygen and Eosin Y. The AFM images (Fig. S2C,D) show the non-drastic morphological changes in the yeast. On comparing the photochemical reaction using *E*. *coli* as negative control, no inhibitory bacteriostatic or bactericidal effect was detected, since a value of 2 × 10^5^ CFU was obtained after the incubation period (see Table 1).

**Figure 5:**
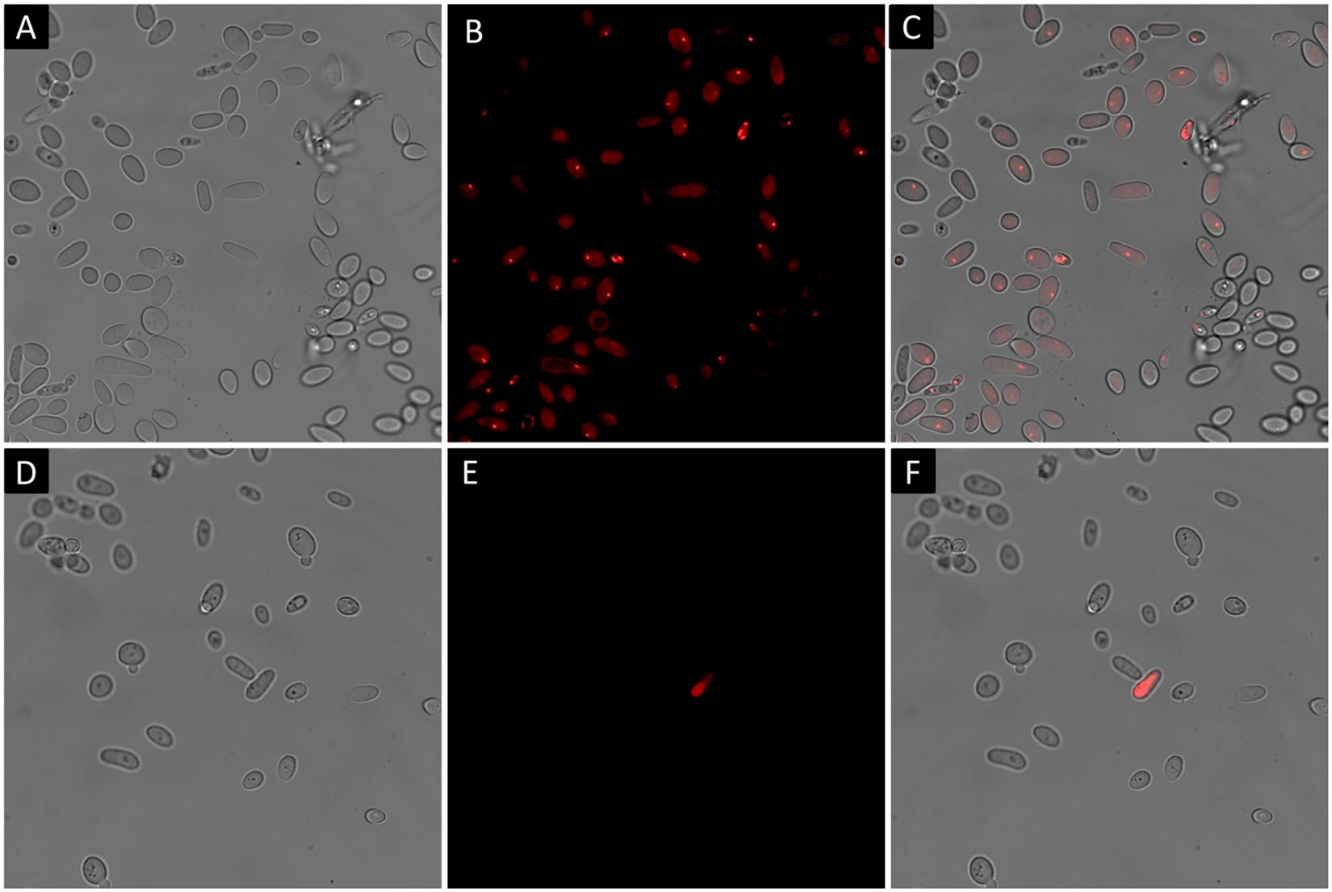
Confocal microscopy images stained with propidium iodide (PI) from *C*. *tropicalis*, showing the differential staining patterns of live and dead yeasts. (**A–C**) Treatment of photosensitization with Eosin Y and light (A = transmitted light image, B = fluorescence image and C = superimposed image). (**D–F**) Control with Eosin Y in dark conditions (D = transmitted light image, E = fluorescence image and F = superimposed image).

If photo-oxidation treatment caused damage to the cell membrane, PI would penetrate the cell due to an increase in permeability, consequently showing an increase in red fluorescence^24^. The cells were examined with a confocal scanning microscope equipped with laser at 543 nm. The confocal microscopy images of the photosensitization treatment of *C*. *tropicalis* and the control under dark conditions are shown in Figs 5 and S3. In the upper part, the images after the photo-oxidation treatment can be seen. The control results under dark conditions are included below. In the photo-oxidation treatment, an increase in red fluorescence in cells is observed due to apparent damage or disruption in the cell membranes as a consequence of the photosensitization treatment. It should be noted that the treated cells were completely stained, which suggests that the fluorochrome easily penetrated inside the cells. These observations are also attributable to damage or significant disturbance within the cell membrane of *C*. *tropicalis*.

## Conclusion

The differences in the morphology of these cell membranes were revealed by means of AFM studies carried out on artificial lipid bilayers, using cholesterol or ergosterol as structural sterol. The lipid bilayers that include ergosterol are apparently destroyed on treatment with singlet oxygen, due to the conversion of ergosterol into its peroxy derivative. Based on these reaction conditions, an antifungal method using *C*. *tropicalis* as model was explored, which provides evidence of membrane permeation under photosensitization. In the fungal cultures subjected to the treatment, no structural damage was detected, but rather an increase in membrane permeability and a total loss of viability. Consequently, photosensitization treatments are a clear and effective antifungal alternative, especially for fungi resistant to azole substances, since they have higher ergosterol concentrations in their membranes. Such structural motifs are ideal candidates for these easy and efficient methods.

## Electronic supplementary material

Supplementary information
