# Supplementary material for: Exploring photosensitization as an efficient antifungal method
Source: Sci Rep. 2018 Sep 27;8:14489. doi: 10.1038/s41598-018-32823-2 (PMC6160477; doi:10.1038/s41598-018-32823-2)
Supplement: Supplementary file 1 — Supplementary information [file 41598_2018_32823_MOESM1_ESM.docx]

**Supplementary Material**

**Exploring photosensitization as an efficient antifungal method**

César Espinoza^1,2^, Miriam C. Rodríguez González^3^, Guillermo Mendoza^1,4^, Alberto Hernández Creus^3^, Ángel Trigos^2^, José J. Fernández ^1,5^*

^1^ Instituto Universitario de Bio-Orgánica Antonio González (IUBO AG), Centro de Investigaciones Biomédicas de Canarias (CIBICAN), Universidad de La Laguna, Universidad de La Laguna (ULL), Avenida Astrofísico Francisco Sánchez 2, 38206 Tenerife, Spain

^2^ Laboratorio de Alta Tecnología de Xalapa, Universidad Veracruzana, Calle Médicos 5, Col. Unidad del Bosque, 91010 Xalapa Enríquez, Veracruz, Mexico

^3^ Área de Química Física, Departamento de Química, Instituto de Materiales y Nanotecnología (IMN), Universidad de La Laguna (ULL), Avenida Astrofísico Francisco Sánchez s.n., 38200 Tenerife, Spain

^4^ Facultad de Ciencias Agrícolas, Universidad Veracruzana, Circuito Gonzalo Aguirre Beltrán s.n., Zona Universitaria, 91090 Xalapa, Veracruz, Mexico

^5^ Departamento de Química Orgánica, Universidad de La Laguna (ULL), Avenida Astrofísico Francisco Sánchez s.n., 38206 Tenerife, Spain


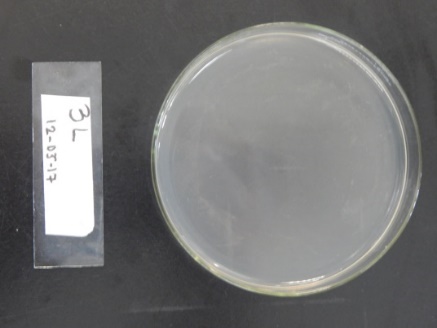

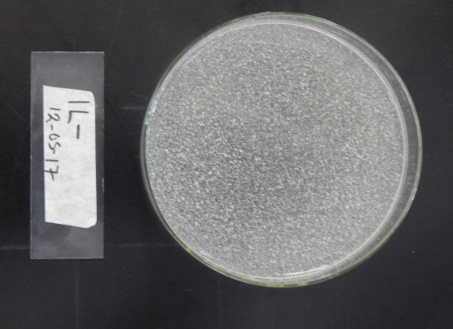

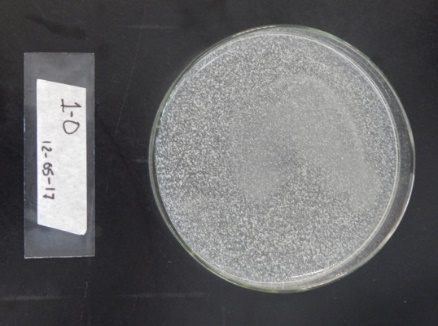

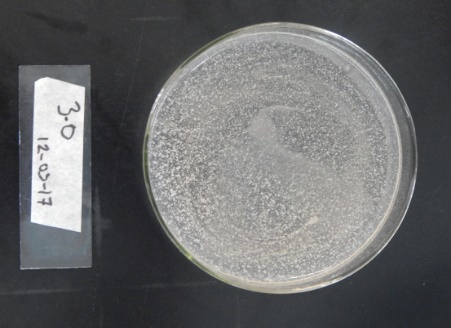


A

B

D

C

**Figure S1.** Cell viability experiments with *C. tropicalis* on PDA medium, 27 ± 2 °C for 72 h. Growth controls: (A) from 1D and (B) from 1L conditions. Photosensitization treatment: (C) negative, from 2D in dark conditions and (D) effective photosensitization treatment, conditions 2L.


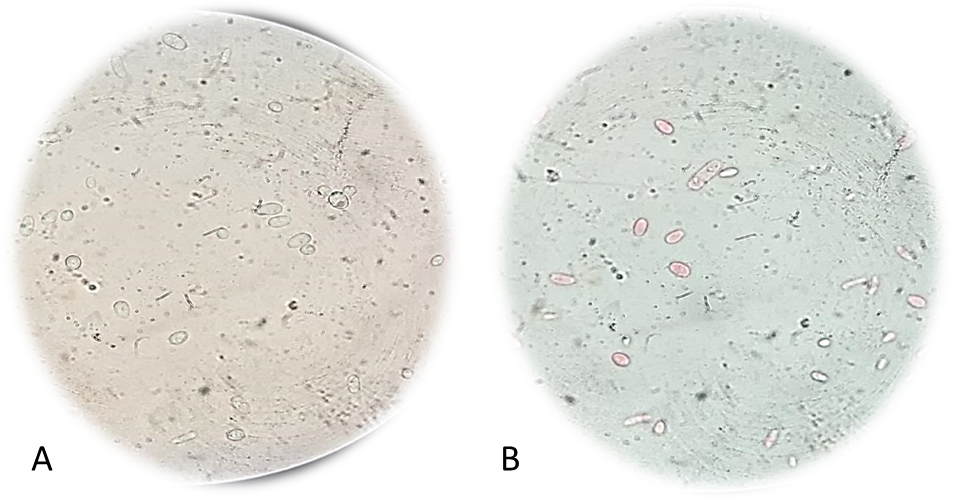

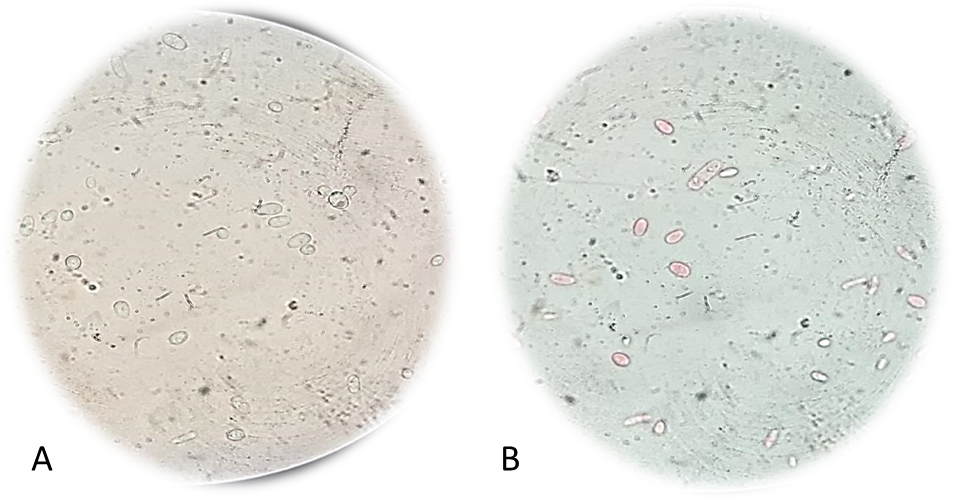


A

B


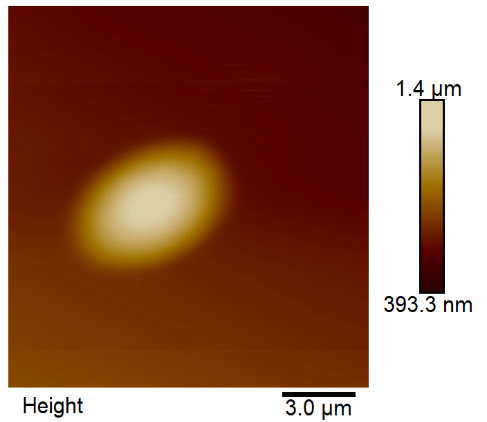

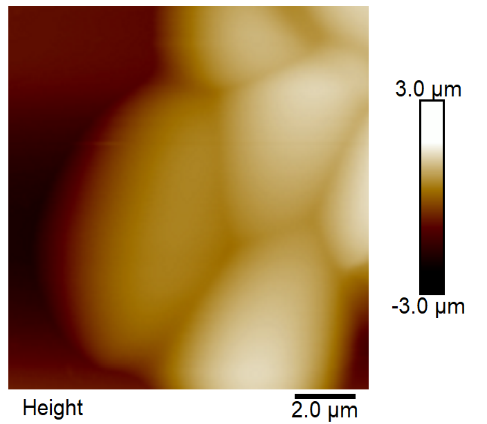


C

D

**Figure S2**. Morphological analysis of *C. tropicalis* by optical microscope 100 X (A) prior to, and (B) after 2L treatment. AFM images (C) and (D) after 2L treatment.

Treatment of photosensitization with Eosin Y and light

Control with Eosin Y in dark conditions

**Figure S3**. Confocal microscopy images stained with propidium iodide (PI) from *C. tropicalis*, showing the different staining patterns of live and dead yeasts. Top: Photosensitization treatment with Eosin Y and light (Left = transmitted light image, Centre = fluorescence image, Right = superimposed image). Bottom: Control with Eosin Y in dark conditions (Left = transmitted light image, Centre = fluorescence image and Right = superimposed image).
